# How to Examine for Life Insurance

**Published:** 1890-09

**Authors:** 


HOW TO EXAMINE FOR LIFE INSURANCE. By JOHN M. KEATING, M. D., President of the Association of Life Insurance Medical Directors, etc. 8vo; pp. viii.—204. Philadelphia: P. Blakiston, Son & Co. 1890.

This is the most practical manual on this subject that has yet been offered as a guide to the medical examiner for life insurance. The author has had a large experience as a medical director of one of the great life insurance companies, and it would, therefore, naturally be expected that he would deal with nothing but the useful and indispensable in a work of this kind. The plates and illustrations are such as contribute to aid diagnosis, and are well executed. Plate III. is especially to be noted for its anatomical and artistic perfection.

It would be well if the decision of the Supreme Court of Pennsylvania, defining the general object of life insurance, quoted in this opening chapter, were printed on every policy. It would disabuse the public mind of a prevalent notion that some principle other than that of indemnity against loss pertains to life insurance.

Every life insurance examiner should possess this book, even though he may be experienced in this work, for it contains much that is needful in the way of reference that cannot be found grouped elsewhere.

